# Moderating effects of plasma glial fibrillary acidic protein along the Alzheimer's disease continuum

**DOI:** 10.1002/alz.70626

**Published:** 2025-09-05

**Authors:** Shannon Y. Lee, Valentina E. Diaz, Olivia M. Emanuel, Emily F. Matusz, Julia Webb, Brandon Chan, Argentina Lario Lago, Wei‐En Wang, Jesse DeSimone, Julio C. Rojas, Lawren VandeVrede, Renaud La Joie, Shellie‐Anne Levy, Franchesca Arias, Brenda A. Wiens, Idaly Velez‐Uribe, Warren W. Barker, Emily W. Paolillo, Mark Sanderson‐Cimino, Monica Rosselli, Sruti Rayaprolu, Tatjana Rundek, Rosie E. Curiel Cid, David E. Vaillancourt, Melissa J. Armstrong, Michael Marsiske, Steven T. DeKosky, David A. Loewenstein, Ranjan Duara, Glenn E. Smith, Adam Staffaroni, Gil D. Rabinovici, Kaitlin B. Casaletto, Joel H. Kramer, Rowan Saloner, Breton M. Asken

**Affiliations:** ^1^ Department of Clinical and Health Psychology University of Florida Gainesville Florida USA; ^2^ Department of Neurology Memory and Aging Center Weill Institute for Neurosciences University of California San Francisco San Francisco California USA; ^3^ 1Florida Alzheimer's Disease Research Center Gainesville Florida USA; ^4^ Department of Applied Physiology and Kinesiology University of Florida Gainesville Florida USA; ^5^ Department of Psychology Florida Atlantic University Boca Raton Florida USA; ^6^ Wien Center for Alzheimer's Disease and Memory Disorders Mt. Sinai Medical Center Miami Beach Florida USA; ^7^ Department of Neurology University of Florida College of Medicine Gainesville Florida USA; ^8^ Departments of Psychiatry and Behavioral Sciences and Neurology Center for Cognitive Neuroscience and Aging University of Miami Miami Florida USA

**Keywords:** Alzheimer's disease, astrocyte reactivity, ATN, cognition, GFAP, glial fibrillary acidic protein, inflammation, neurodegeneration, neuroinflammation, neuropathology, plasma biomarkers, plasma GFAP

## Abstract

**INTRODUCTION:**

Glial fibrillary acidic protein (GFAP) may contribute to Alzheimer's pathology at early disease stages. GFAP moderation of Alzheimer's disease (AD)‐related neurodegeneration and cognition is unclear.

**METHODS:**

We examined plasma GFAP moderation of AD biomarkers (amyloid beta [Aβ]‐positron emission tomography [PET][A]; plasma phosphorylated tau‐181 [p‐tau181][T_1_]), neurodegeneration (plasma NfL[N_plasma_]; structural magnetic resonance imaging [MRI][N_MRI_]), and cognition (Cog_memory_; Cog_executive_) in two cohorts: University of California San Francisco (UCSF) (*N* = 212, 91.0% non‐Hispanic/Latino White [NHLW], age = 74.7 [7.6] years, 75.9% cognitively unimpaired [CU]) and 1Florida Alzheimer's Disease Research Centers (1FLADRC; *N* = 582, 32.8% NHLW, age = 70.7 [8.5] years, 28.9% CU).

**RESULTS:**

Plasma GFAP consistently moderated A–T_1_ (UCSF: *β* = 0.46, *p* = 0.012; 1FLADRC: *β* = 0.12, *p *= 0.029). The association between elevated Aβ‐PET and increased (p‐tau) was strengthened at higher GFAP concentrations. In 1FLADRC, GFAP moderated T_1_–N_plasma/MRI._ In UCSF, GFAP moderated T_1_–Cog_memory/executive_ and N_MRI_–Cog_memory/executive_. Higher GFAP consistently related to worse neurodegeneration and cognition (main effects).

**DISCUSSION:**

Across demographically and clinically heterogeneous cohorts, plasma GFAP is a key moderator of AD and may help identify individuals at greatest risk of AD‐related neurodegeneration and cognitive decline.

**Highlights:**

- AD biomarkers were measured in two demographically and clinically distinct cohorts.
- Plasma GFAP moderated Aβ‐PET to p‐tau associations in both UCSF and 1FLADRC.
- Cohort‐dependent, GFAP moderated p‐tau to neurodegeneration and cognition associations.
- All moderations revealed strengthened disease associations with higher plasma GFAP.
- Plasma GFAP may help identify individuals at greatest risk of AD‐related decline.

## 1 BACKGROUND

Alzheimer's disease (AD) is neuropathologically defined by the accumulation of amyloid beta (Aβ) plaques and tau neurofibrillary tangles. Proposed biological frameworks for AD, such as AT(N) highlight the interplay of Aβ (A) and tau (T) upstream of neurodegeneration (N) and subsequent cognitive decline.^1^, ^2^ However, there is significant variability in AD progression and the degree to which AD pathology translates to neurodegeneration and cognitive decline. More recent frameworks recognize important biological contributors to AD and dementia beyond AT(N), including inflammation (I) and non‐AD vascular (V) and α‐synuclein (S) co‐pathologies in AT‐NI‐VS.^3^

Inflammation is a non‐specific neurobiological process implicated in AD pathophysiology.^3^, ^4^ A key source of neuroinflammation is astrocytes, which become reactive and undergo functional and morphological changes in the presence of proteotoxicity from AD and other neurodegenerative diseases.^5^, ^6^, ^7^ Functionally, astrocyte reactivity is often accompanied by a pro‐inflammatory shift that may initially be protective but can exacerbate neuroinflammation or neuronal injury when chronic.^8^, ^9^ Astrocyte reactivity upregulates glial fibrillary acidic protein (GFAP), a cytoskeletal protein essential for maintaining astrocyte structure and integrity.^10^ Recent fluid biomarker, neuropathological, and animal research in AD suggests that more severe astrocyte reactivity may contribute to propagating Aβ‐dependent tau phosphorylation at early disease stages^11^, ^12^, ^13^ and that GFAP‐positive astrocytes can internalize tau and may be implicated in its further spread.^14^, ^15^

Blood‐based GFAP biomarkers provide a minimally invasive and more widely accessible means of capturing a component of inflammation relevant to AD.^16^, ^17^ In AD studies, plasma GFAP has enabled complementary measurement alongside other established AD biomarkers in vivo.^18^, ^19^ Recently, a large study of 1016 cognitively unimpaired (CU) adults found that elevated levels of plasma GFAP related to strengthened relationships between Aβ‐positron emission tomography (PET) and both phosphorylated tau fluid biomarkers and tau PET deposition.^11^ Such findings suggest potential for targeting astrocyte reactivity during preclinical stages of AD to mitigate cognitive decline. However, it is unknown whether plasma GFAP levels moderate the effects of tau phosphorylation on neurodegeneration and cognition. This could inform more precise clinical risk stratification, enrich clinical trial enrollment, and create opportunities for earlier intervention for individuals at greatest clinical risk. Moreover, it is also unclear whether these relationships relate to the stage of clinical severity and person‐specific factors such as biological sex and ethnoracial background.

We studied two independent cohorts from the University of California San Francisco (UCSF) and 1Florida Alzheimer's Disease Research Centers (1FLADRC) across the AD and cognitive continuum to investigate the effects of astrocyte reactivity (plasma GFAP) on different components of the AD cascade, including Aβ pathology (Aβ‐PET), phosphorylated and secreted AD tau (plasma phosphorylated tau [p‐tau]181 and p‐tau217), neurodegeneration (brain MRI, plasma neurofilament light [NfL]), and cognition (memory, executive functioning).

## 2 METHODS

### 2.1 Participants

Data for this multicohort study were collected between February 2014 and January 2023 across two sites, including the UCSF Brain Aging Network for Cognitive Health and ADRC and 1FLADRC. UCSF participants were community‐dwelling older adults with normal cognition or mild cognitive impairment (MCI). 1FLADRC participants spanned the continuum of normal cognition, MCI, and dementia and were recruited from outpatient memory disorders clinics, free memory screening programs, and community outreach. The UCSF sample was predominantly non‐Hispanic/Latino White, while over 50% of 1FLADRC participants self‐identified as Hispanic/Latino White. Participants from both cohorts completed neurological and neuropsychological evaluations, including the National Alzheimer's Coordinating Center (NACC) Uniform Data Set (UDS) plus cognitive measures specific to each center. Overall functioning was assessed with the Clinical Dementia Rating (CDR) scale. Study protocols were approved by the respective site Institutional Review Boards, and each participant provided written informed consent to study procedures.

### 2.2 Plasma biomarkers

Venous blood was collected and processed following established and standardized protocols prior to analysis in duplicate (Supplementary Methods). Single‐molecule array (Simoa) technology was utilized for p‐tau181 (Quanterix p‐tau181 Advantage V2), p‐tau217 (ALZpath, 1FLADRC only), GFAP, and NfL (UCSF: Quanterix Neurology 4‐Plex A; 1FLADRC: Quanterix Neurology 2‐Plex B). Both cohorts analyzed plasma p‐tau181 with the Quanterix p‐tau181 Advantage V2 assay. The scale of raw concentration ranges differs between cohorts due to an update to the assay that occurred between the time each cohort's samples were analyzed (UCSF in 2021 and 1FLADRC in 2023). In both cohorts, we only included sample concentrations with coefficients of variance (CV) < 20%. All plasma biomarker data were log_10_‐transformed for statistical analysis.

**RESEARCH IN CONTEXT**

1. **Systematic review**: Astrocyte reactivity, measured via plasma GFAP, contributes to amyloid‐related tau pathology at early stages of AD. It is less clear whether plasma GFAP modulates AD‐related neurodegeneration and the severity of cognitive deficits.
2. **Interpretation**: Plasma GFAP moderated Aβ‐PET to plasma p‐tau181/217 associations across two demographically and clinically distinct cohorts. GFAP exhibited cohort‐dependent moderation of p‐tau to neurodegeneration (1FLADRC) and p‐tau and neurodegeneration to cognition relationships (UCSF). In all cases, disease associations strengthened with higher plasma GFAP. Astrocyte reactivity may more prominently and consistently influence earlier AD pathology than downstream effects on neurodegeneration and cognitive decline.
3. **Future directions**: Plasma GFAP supports the identification of individuals most at risk of AD‐related neurodegeneration and cognitive decline, suggesting utility for risk stratification, trial enrollment, and earlier intervention. Replication in ethnoracially representative cohorts and longitudinal follow‐up will improve understanding of temporal plasma GFAP, AD biomarker, neurodegeneration, and cognition associations.

### 2.3 Structural neuroimaging

Participants underwent 3T structural magnetic resonance imaging (MRI) at UCSF (Siemens Trio Tim or Prisma Fit) or 1FLADRC (Siemens Magnetom Skyra, Vida, or Prisma). All T1‐weighted images were acquired via magnetization‐prepared rapid gradient‐echo (MPRAGE) sequences and a standard acquisition protocol (Supplementary Methods). In UCSF, T1 scans were processed using Diffeomorphic Anatomical Registration using Exponentiated Lie algebra (DARTEL) in Statistical Parametric Mapping (SPM) for voxel‐based morphometry (VBM).^20^ In 1FLADRC, T1 scans were processed using FreeSurfer (version 7.3.2) in SPM for surface‐based morphometry (SBM).^21^, ^22^ The primary outcome was the aggregate volume of 10 bilateral AD‐prone regions of interest (ROIs) (AD meta‐ROI)^23^, ^24^: hippocampus, entorhinal cortex, parahippocampal gyrus, amygdala, fusiform gyrus, precuneus, and temporal pole and middle temporal, inferior temporal, and inferior parietal cortices.

### 2.4 Amyloid PET

Aβ‐PET was performed in subsets of participants with either [18F] florbetaben or [18F] florbetapir (UCSF: *N* = 129; 1FLADRC: *N* = 264; Supplementary Methods). For quantification, global composite standardized uptake value ratios (SUVRs; whole cerebellum reference) were independently calculated and converted to Centiloid (CL) scales at each site.^25^ Aβ‐PET scans were classified as either positive or negative via trained readers blinded to quantification.

### 2.5 Cognitive assessment

Participants underwent comprehensive neuropsychological testing. We created composite scores for memory and executive functioning that allowed for harmonizing shared (NACC UDS) and site‐specific tests on the same scale (Supplementary Methods).^26^, ^27^ Higher memory and executive functioning composite scores indicate better performance.

### 2.6 Model terms

We utilized the updated Alzheimer's Association framework for AD staging and diagnosis to define our biomarker measures,^3^ which divides (*T*) biomarkers into *T*_1_ and *T*_2_ subcategories, with T1 biomarkers including plasma p‐tau epitopes (181, 217, 231).^2^ (A) and *T*_1_ are “Core 1 biomarkers” reflecting early AD biomarker changes^3^ and are the focus of this study. We defined biomarker and cognitive measures as: astrocyte reactivity (plasma GFAP), Aβ (A; dichotomously based on Aβ‐PET visual read [A_VR;_ Aβ+ vs Aβ−] and continuously as global cortical Aβ burden in CLs [A_CL_]); phosphorylated tau (T_1;_ plasma p‐tau181 [both cohorts] or p‐tau217 [1FLADRC]); neurodegeneration (N; AD meta‐ROI volume [N_MRI_] or plasma NfL [N_plasma_]); cognition (Cog; memory [Cog_memory_] or executive function [Cog_executive_]).

### 2.7 Statistical analysis

All analyses were performed using RStudio version 2024.04.1. Pearson correlations examined bivariate associations of plasma GFAP with primary AT_1_(N) biomarkers and cognition. Unconditional relationships (i.e., without GFAP moderation) between AD biomarkers (A–*T*_1_), measures of neurodegeneration (*T*_1_–N_MRI_, *T*_1_–N_plasma_), and cognition (*T*_1_–Cog_memory,_ *T*_1_–Cog_executive,_ N_MRI_–Cog_memory,_ N_MRI_–Cog_executive_, N_Plasma_–Cog_memory,_ N_Plasma_–Cog_executive_) were assessed using linear regression.

To examine the moderating role of astrocyte reactivity, we entered an interaction term between plasma GFAP and the primary predictor for each model. We examined all pairwise associations except for A‐to‐N and A‐to‐Cog since we did not hypothesize an unconditional main effect of these relationships.^28^, ^29^, ^30^, ^31^, ^32^, ^33^, ^34^ For models with statistically significant moderation, we applied Benjamini–Hochberg false discovery rate (FDR)^35^ correction for multiple comparisons. Reported *p* values throughout the results are uncorrected and survived FDR correction unless otherwise noted. For significant moderation effects, we employed the Johnson–Neyman^36^, ^37^ method to identify specific boundaries (regions of significance) of plasma GFAP levels at which A*T*_1_(N) and cognition associations were statistically significant. Covariates included age, sex, *APOE* ε4 carrier status, intracranial volume, scanner (MRI models), and education (cognition models). For all interaction models where plasma GFAP moderation was not observed, interaction terms were removed, and the main effects of GFAP were tested.

Post hoc analyses used three‐way interactions to examine demographic effects (sex, both cohorts; ethnoracial group; 1FLADRC only) on GFAP moderation (e.g., Ab‐PET × GFAP × sex). Significant three‐way interactions were probed via demographic‐stratified models. Ethnoracial differences within Aβ‐PET models were evaluated in Hispanic/Latino White and non‐Hispanic/Latino White groups. Lastly, analyses were repeated in 1FLADRC using plasma p‐tau217, which typically demonstrates stronger correspondence with AD pathology than p‐tau181.^38^

## 3 RESULTS

### 3.1 Participant characteristics

This study included 212 participants from UCSF (age 74.7 ± 7.6 years, 50.5% female, 91.0% non‐Hispanic/Latino White, 78.5% CU; *N* = 129 with Aβ‐PET, 27.9% Aβ‐PET+) and 582 participants from 1FLADRC (age 70.7 ± 8.5 years, 56% female, 32.8% non‐Hispanic/Latino White, 28.9% CU, *N* = 264 with Aβ‐PET, 40.5% Aβ‐PET+; Table 1). UCSF participants were significantly older than 1FLADRC (74.7 ± 7.6 vs 70.7 ± 8.5; *p* < 0.001) and included fewer individuals with cognitive impairment (UCSF: 22% CDR Global = 0.5; 1FLADRC: 71% CDR Global ≥ 0.5). In UCSF, 63.6% of MCI participants with Aβ‐PET were amyloid positive. In 1FLADRC, 39.7% of MCI and 76.2% of dementia participants with Aβ‐PET were amyloid positive, respectively (Supplementary Results Table S1).

**TABLE 1:** Participant characteristics by study cohort

	UCSF	1FLADRC
***n***	212	582
Age, years, mean (SD)	74.7 (7.6)	70.7 (8.5)
Education, years, mean (SD)	17.6 (2.2)	15.5 (8.6)
Sex, *N* (% female) = 2 (%)	107 (50.5)	326 (56.0)
Ethnoracial GROUP, *N*%		
Non‐Hispanic/Latino White	193 (91.0)	191 (32.8)
Hispanic/Latino White^*^	N/A	245 (42.1)
Black/African American	2 (1.0)	131 (22.5)
Other/not reported	17 (8.0)	15 (2.6)
APOE ε4, *N* (% carrier)	65 (30.7)	185 (31.8)
Global CDR, *N* (%)		
0	161 (75.9)	168 (28.9)
0.5	44 (20.8)	333 (57.2)
1+	0 (0.0)	81 (13.9)
Not reported	7 (3.3)	N/A
Memory composite	0.61 (0.20, 0.85)	0.33 (−0.28, 0.91)
Executive composite	0.21 (−0.36, 0.71)	−1.29 (−2.21, −0.54)
Plasma p‐tau181 (pg/mL)^†^	1.52 (1.12, 2.15)	23.2 (17.3, 33.5)
Plasma GFAP (pg/mL)	152 (111, 206)	160 (111, 248)
Plasma NfL (pg/mL)	21.6 (15.9, 28.7)	13.8 (9.2, 20.8)
Aβ‐PET (+), N (%)^‡^	36 (27.9)	107 (40.5)
Aβ‐PET Centiloids (CL)	21.3 (40.8)	29.5 (38.6)

* Hispanic/Latino White participants in 1FLADRC (n = 245) reported the following ethnic origins: 140 (57.1%) Cuban, 76 (31.0%) South American, 11 (4.5%) Puerto Rican,18 (7.4%) Other. Hispanic/Latino ethnic origin information was not available for the UCSF cohort.

^†^ Different assay versions in each cohort with resulting different magnitude/scale of values.

^‡^ *N* with Aβ‐PET (UCSF) = 129, N with Aβ‐PET (1FLADRC) = 264.

*Note*: all values are reported as median (IQR), unless otherwise noted; IQR = interquartile range; SD = standard deviation.

Abbreviations: 1FLADRC, 1Florida Alzheimer's Disease Research Centers; APOE, apolipoprotein E; CDR, Clinical Dementia Rating scale; GFAP, glial fibrillary acidic protein; NfL, neurofilament light; UCSF, University of California San Francisco.

In both UCSF and 1FLADRC, correlations between plasma GFAP and AT_1_(N) biomarkers and cognitive outcomes showed positive associations between A_CL_–GFAP, *T*_1_–GFAP, and N_Plasma_–GFAP and negative associations between N_MRI_​ –GFAP, Cog_memory_​–GFAP, and Cog_executive_​​–GFAP (Supplementary Results Table S2). Unconditional models testing main effects of AT_1_(N) and cognition without GFAP showed positive associations between A–*T*_1,_ *T*_1_–N_Plasma_, and N_MRI_–Cognition and negative relationships between *T*_1_–N_MRI,_ *T*_1_–Cognition, and N_Plasma,_–Cognition (Supplementary Results Table S3).

### 3.2 Moderation analyses

#### 3.2.1 A–T_1_: Plasma GFAP moderation of Aβ‐PET and plasma p‐tau181 relationship

**FIGURE 1:**
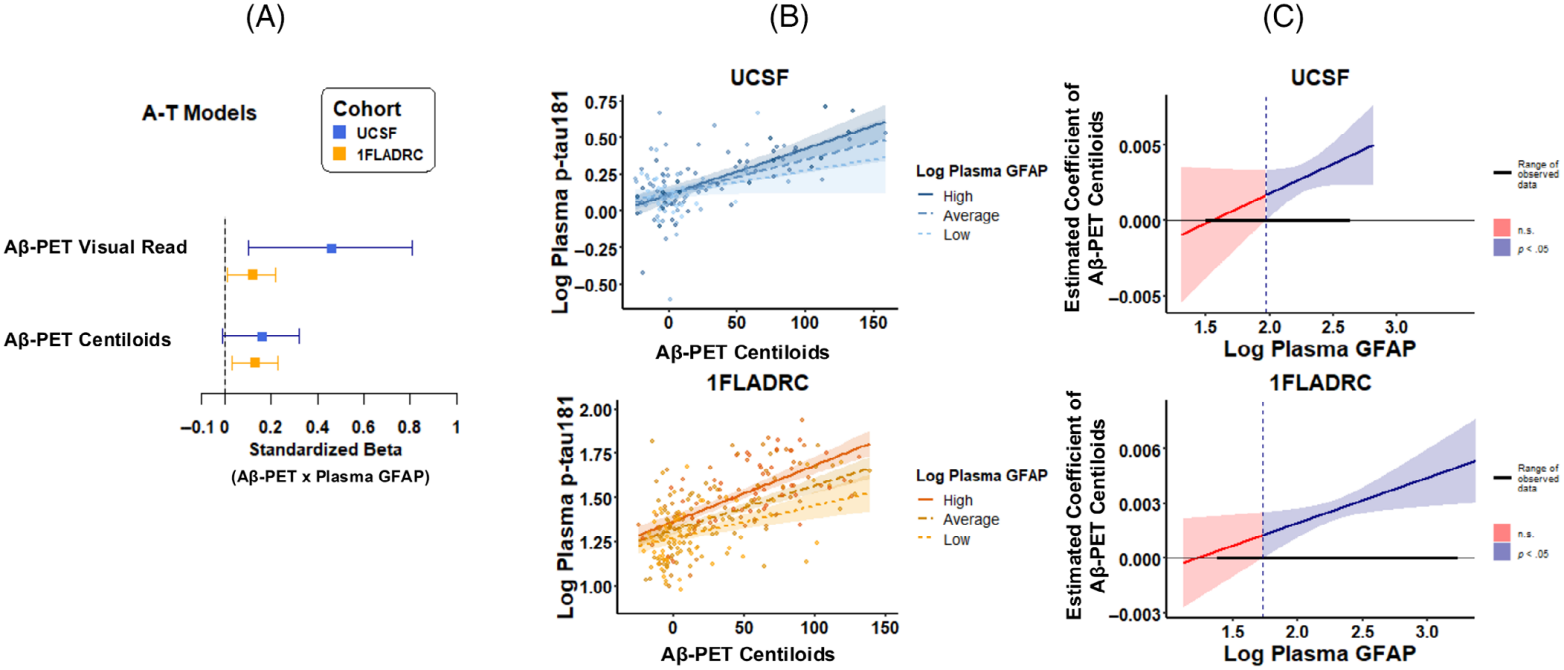
Plasma GFAP moderating effects of Aβ‐PET and plasma p‐tau181 relationships. Aβ‐PET was performed in a subset of participants in each cohort (UCSF: *N* = 129; 1FLADRC: *N* = 264). (A) values represent standardized coefficients with bootstrapped 95% confidence intervals for the interaction of Aβ‐PET (visual read or Centiloids) and plasma GFAP on plasma p‐tau181, reported by cohort. (B) Plasma GFAP moderation of Aβ‐PET (Centiloids) and plasma p‐tau181 stratified by high (+1 SD), average (mean), and low (−1 SD) levels of plasma GFAP. (C) The effect size (unstandardized) of Aβ‐PET on plasma p‐tau181 is plotted across plasma GFAP levels. Blue regions represent levels of plasma GFAP at which the relationship between Aβ‐PET (Centiloids) and plasma p‐tau181 is statistically significant. 1FLADRC, 1Florida Alzheimer's Disease Research Center; GFAP, glial fibrillary acidic protein; UCSF, University of California San Francisco.

Plasma GFAP significantly moderated the relationship between Aβ‐PET (A_VR_) and plasma p‐tau181 in both UCSF (β [95% CI] = 0.46 [0.10, 0.81]; *p = 0*.012) and 1FLADRC (β [95% CI] = 0.12 [0.01, 0.22]; *p *= 0.029), such that the positive association between Aβ‐PET and plasma p‐tau181 increased in magnitude with higher plasma GFAP (Figure 1). J–N analyses suggested that Aβ‐PET positivity was significantly related to higher plasma p‐tau181 above a plasma GFAP threshold of 98 pg/mL in UCSF and 57 pg/mL in 1FLADRC (Figure 1, Supplementary Results Table S4). The GFAP threshold in both cohorts is low, suggesting that Aβ‐PET relates to higher p‐tau181 irrespective of GFAP concentration, but the association is significantly stronger at higher GFAP concentrations. GFAP moderation of the association between Aβ‐PET and plasma p‐tau181 was also observed when measuring Aβ burden via CLs in 1FLADRC (β [95% CI] = 0.13 [0.03, 0.23]; *p *= 0.013; J–N threshold > 54 pg/mL). A similar effect was observed in UCSF (β [95% CI] = 0.16 [−0.01, 0.32]; *p = *0.06; J–N threshold 94 pg/mL).

#### 3.2.2 T_1_–N: Plasma GFAP moderation of plasma p‐tau181 and neurodegeneration relationship

**FIGURE 2:**
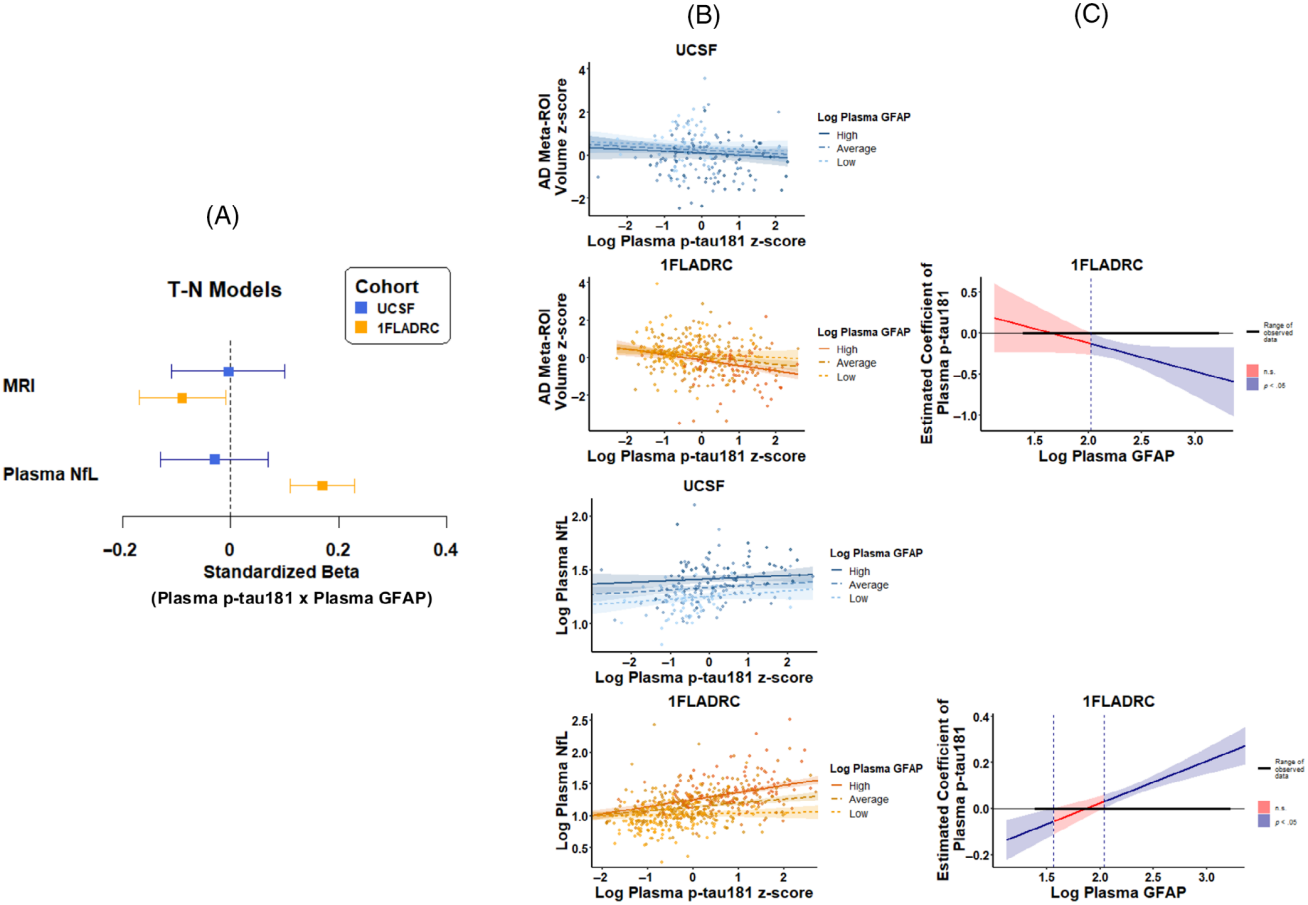
Plasma GFAP moderating effects of plasma p‐tau181 and neurodegeneration (AD meta‐ROI and plasma NfL) relationships. (A) values represent standardized coefficients with bootstrapped 95% confidence intervals for the interaction of plasma p‐tau181 and plasma GFAP on AD meta‐ROI or plasma NfL, reported by cohort. (B) Plasma GFAP moderation of plasma ptau‐181 and AD meta‐ROI or plasma NfL, stratified by high (+1 SD), average (mean), and low (−1 SD) levels of plasma GFAP. (C) Effect size (unstandardized) of p‐tau181 on neurodegeneration is plotted across plasma GFAP levels. Blue regions represent levels of plasma GFAP at which the relationship between p‐tau181 and neurodegeneration is statistically significant. AD, Alzheimer's disease; GFAP, glial fibrillary acidic protein; NfL, neurofilament light; ROI, region of interest.

In 1FLADRC only, plasma GFAP significantly moderated the relationship between plasma p‐tau181 and plasma NfL; (T_1_–N_Plasma_: β [95% CI] = 0.17 [0.11, 0.23]; *p *< 0.0001; J–N threshold > 108 pg/mL; Figure 2, Supplementary Results Table S4), such that the association between higher plasma p‐tau181 and increased plasma NfL only emerged at GFAP levels > 100 pg/mL. Plasma GFAP also moderated the relationship between plasma p‐tau181 and AD meta‐ROI volume (*T*_1_–N_MRI_: β [95% CI] = −0.09 [−0.17, −0.01]; J–N threshold > 103 pg/mL), but the association did not survive FDR correction (uncorrected *p = *0.040; FDR‐corrected *p = 0*.081). In UCSF, plasma GFAP did not significantly moderate the relationship between plasma p‐tau181 and neurodegeneration measures *(ps >* *0*.05), though main effects of higher plasma GFAP and increased neurodegeneration were observed independently of plasma p‐tau181 (GFAP N_MRI_: β [95% CI] = −0.14 [−0.26, −0.02], *p *= 0.022; p‐tau181 N_MRI_: β [95% CI] = −0.08 [−0.20, 0.03], *p > *0.05; GFAP N_Plasma_: β [95% CI] = 0.43 [0.31, 0.56]; *p *< 0.001; p‐tau181 N_Plasma_: β [95% CI] = 0.10 [‐0.01, 0.22], *p > *0.05).

#### 3.2.3 T_1_–Cognition: Plasma GFAP moderation of plasma p‐tau181 and cognition relationship

**FIGURE 3:**
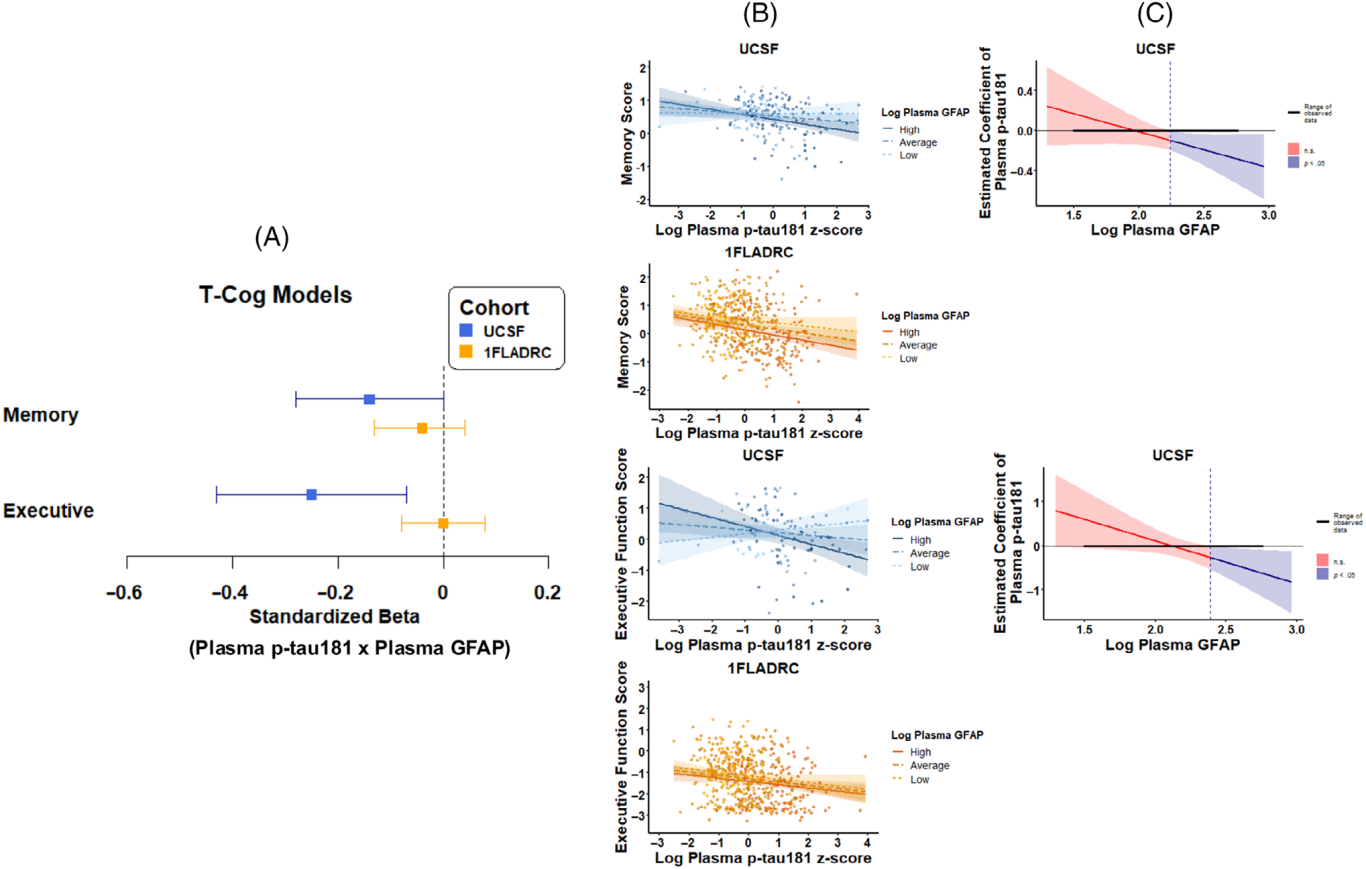
Plasma GFAP moderating effects of plasma p‐tau181 and cognition (memory and executive function) relationships. (A) Values represent standardized coefficients with bootstrapped 95% confidence intervals for the interaction of plasma p‐tau181 and plasma GFAP on cognition, reported by cohort. (B) plasma GFAP moderation of plasma ptau‐181 and memory or executive function, stratified by high (+1 SD), average (mean), and low (−1 SD) levels of plasma GFAP. (C) The effect size (unstandardized) of p‐tau181 on cognition is plotted across plasma GFAP levels. Blue regions represent levels of plasma GFAP at which the relationship between p‐tau181 and cognition is statistically significant. GFAP, glial fibrillary acidic protein.

In UCSF only, plasma GFAP significantly moderated the relationship between plasma p‐tau181 and executive functioning (T_1_–Cog_executive_: β [95% CI] = −0.25 [−0.43, −0.07]; *p *= 0.007; J–N threshold > 200 pg/mL; Figure 3, Supplementary Results Table S4), such that higher plasma p‐tau181 was only associated with worse executive function when GFAP levels exceeded 200 pg/mL. Plasma GFAP also moderated the relationship between plasma p‐tau181 and memory (*T*_1_–Cog_memory_: β [95% CI] = −0.14 [−0.28, 0.00]; J–N threshold > 155 pg/mL), but the association did not survive FDR correction (uncorrected *p *= 0.044; FDR‐corrected *p *= 0.089).

In 1FLADRC, plasma GFAP did not moderate the relationship between plasma p‐tau181 and memory or executive function (*p*s > 0.05), though the main effects of higher plasma GFAP and worse cognition were observed independently of plasma p‐tau181 (GFAP Cog_memory_: β [95% CI] = −0.22 [−0.33, −0.12], *p *< 0.001; p‐tau181 Cog_memory_: β [95% CI] = −0.18 [−0.28, −0.09], *p *< 0.001; GFAP Cog_executive_: β [95% CI] = −0.12 [−0.23, −0.02], *p = 0*.021; p‐tau181 Cog_executive_: β [95% CI] = −0.15 [−0.24, −0.05], *p < *0.001).

#### 3.2.4 N–Cognition: Plasma GFAP moderation of neurodegeneration and cognition relationship

**FIGURE 4:**
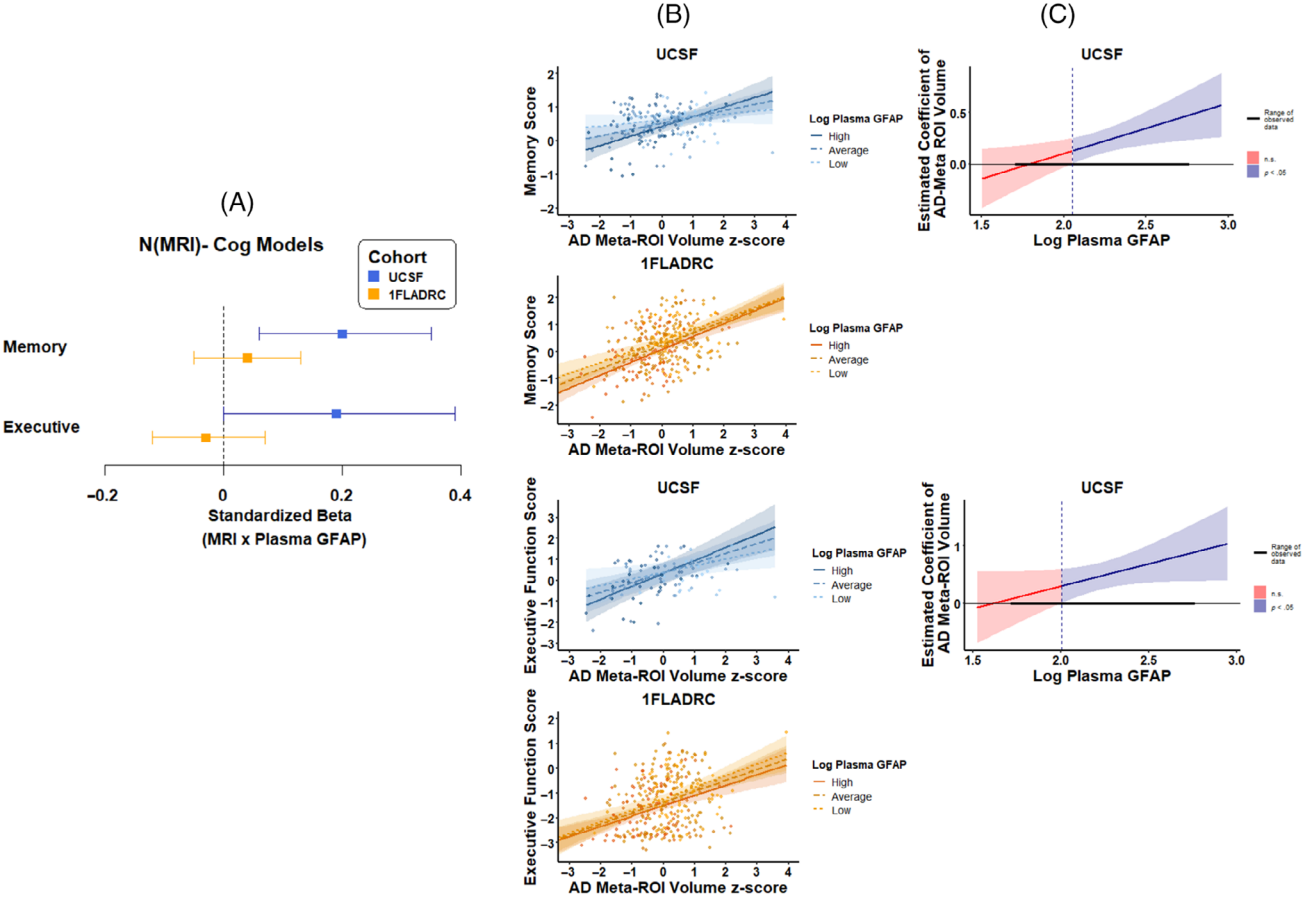
Plasma GFAP moderating effects of neurodegeneration (AD meta‐ROI) and cognition (memory and executive function) relation. (A) Values represent standardized coefficients with bootstrapped 95% confidence intervals for the interaction of AD meta‐ROI and plasma GFAP on cognition, reported by cohort. (B) Plasma GFAP moderation of AD meta‐ROI and memory or executive function, stratified by high (+1 SD), average (mean), and low (−1 SD) levels of plasma GFAP. (C) The effect size (unstandardized) of AD meta‐ROI on cognition is plotted across plasma GFAP levels. Blue regions represent levels of plasma GFAP at which the relationship between neurodegeneration (AD meta‐ROI) and cognition is statistically significant. AD, Alzheimer's disease; GFAP, glial fibrillary acidic protein; ROI, region of interest.

In UCSF only, plasma GFAP significantly moderated the relationship between AD meta‐ROI volume and both memory (N_MRI_–Cog_memory_: β [95% CI] = 0.20 [0.06, 0.35], *p = *0.0064; J–N threshold > 110 pg/mL) and executive function (N_MRI_–Cog_executive_: β [95% CI] = 0.19 [0.00, 0.39], *p = *0.048; J–N threshold > 98 pg/mL; Figure 4, Supplementary Results Table S4), such that the association between lower AD meta‐ROI volume and worse cognition only emerged when plasma GFAP exceeded ∼100 pg/mL. In 1FLADRC, plasma GFAP did not moderate the relationship between AD meta‐ROI volume or memory or executive function (*p*s > 0.05), though the main effects of higher plasma GFAP and worse cognition were observed independently of AD meta‐ROI volume (GFAP Cog_memory_: β [95% CI] = −0.18 [−0.29, −0.08], *p *< 0.001; AD meta‐ROI Cog_memory_: β [95% CI] = 0.54 [0.42, 0.66], *p *< 0.001; GFAP Cog_executive_: β [95% CI] = −0.16 [−0.27, −0.05], *p = 0*.02; AD meta‐ROI Cog_executive_: β [95% CI] = 0.41 [0.28, 0.53], *p < *0.001).

Plasma GFAP did not moderate the relationship between plasma NfL and cognition in either cohort (*ps *> 0.05). The main effects of higher GFAP and worse cognition were observed independently of plasma NfL in 1FLADRC (GFAP Cog_memory_: β [95% CI] = −0.26 [−0.37, −0.16], *p *< 0.001; NfL Cog_memory_: β [95% CI] = −0.07 [−0.18, 0.03], *p *> 0.05; GFAP Cog_executive_: β [95% CI] = −0.12 [−0.23, −0.01]; *p = 0*.024; NfL Cog_executive_: β [95% CI] = −0.13 [−0.24, −0.03]; *p = *0.01) but not UCSF *(ps *> 0.05).

### 3.3 Post hoc analyses

#### 3.3.1 Demographic effects

We employed three‐way interactions to examine the effects of sex (both UCSF and 1FLADRC) and ethnoracial group (1FLADRC only) on plasma GFAP moderation (Supplementary Results Tables S5 and S6). In 1FLADRC, there was a significant three‐way interaction of plasma p‐tau181 × plasma GFAP × sex on plasma NfL (N_plasma_: β [95% CI] = 0.09 [0.03, 0.16], *p *= 0.003) and memory (Cog_memory_: β [95% CI] = −0.13 [−0.25, −0.01], *p *= 0.023). Sex stratification suggested that the moderating effect of plasma GFAP was only significant in females (*T*_1_‐N_plasma_: Females—β [95% CI] = 0.26 [0.18, 0.34], *p *< 0.001; Males—β [95% CI] = 0.07 [Ȓ0.03, 0.16], *p *= 0.18; T_1_‐Cog_memory_: Females—β [95% CI] = −0.12 [−0.24, 0.00], *p *= 0.030; Males—β [95% CI] = 0.04 [−0.07, 0.16], *p *= 0.46).

There was a significant three‐way interaction of plasma p‐tau181 x plasma GFAP x ethno‐racial group on plasma NfL (N_plasma_: β [95% CI] = 0.20 [0.05, 0.26], *p *= 0.01), such that the moderating effect of plasma GFAP was only significant in Hispanic/Latino White participants (*T*_1_‐N_plasma_: HW—β [95% CI] = 0.24 [0.16 0.32], *p <* *0*.001; NHW–β [95% CI] = 0.03 [−0.10, 0.16], *p *= 0.61).

#### 3.3.2 Plasma p‐tau217 replication

Analyses were repeated in 1FLADRC using plasma p‐tau217. All results from p‐tau181 models were replicated with p‐tau217 and generally were of greater magnitude (Supplementary Results Table S7).

## 4 DISCUSSION

We examined whether astrocyte reactivity, proxied by plasma GFAP, moderated AD pathology biomarkers and their associations with neurodegeneration and cognition. We presented multicohort in vivo evidence that relationships between core AD biomarkers, neurodegeneration, and cognition often depend on plasma GFAP concentration. These data suggest that reducing astrocyte reactivity may protect against AD pathological burden at different disease stages. Our findings further support the importance of considering plasma GFAP within the biological framework of AD.

Plasma GFAP moderated the association between cortical amyloid and plasma phosphorylated tau (p‐tau181 and p‐tau217), a robust finding across UCSF and 1FLADRC cohorts and other studies.^11^, ^39^ This extends prior work showing plasma GFAP mediates Aβ‐PET effects on plasma p‐tau and tau‐PET burden^40^, ^41^ and aligns with animal models linking astrocyte reactivity to tau pathology.^42^, ^43^ Our group‐level observations are exemplified in 1FLADRC by a 69‐year‐old male with MCI, who had high Aβ‐PET burden (CL = 101) but lower than expected plasma p‐tau217 (0.42 pg/mL; internal positivity cutoff = 0.56 pg/mL^29^) and GFAP (64 pg/mL, sample mean of Aβ‐PET positive = 257 pg/mL). As cortical amyloid deposition and plasma p‐tau elevations are among the earliest detectable markers of AD pathology, astrocyte reactivity may represent a promising target for early intervention^11^ or explain, in part, cases with discordant AD biomarkers (e.g., Aβ‐PET positive, plasma p‐tau negative).^44^

Reactive astrocytes are present across multiple neurodegenerative diseases, but plasma GFAP correlates more consistently with AD neuropathology (particularly brain amyloid) than other proteinopathies or cerebrovascular disease.^45^, ^46^, ^47^, ^48^ Our study extends prior work by evaluating in vivo astrocyte reactivity as a moderator of presumed later‐disease phenomena, including neurodegeneration and cognitive symptoms. These associations were less consistent across cohorts. In UCSF, representing on average earlier disease and milder symptoms, plasma GFAP moderated associations of plasma p‐tau181 and neurodegeneration with cognition (memory). In contrast, the lack of cognitive associations in 1FLADRC may reflect more non‐AD contributions to cognition that emerge with more severe cognitive impairment (i.e., mixed pathology). It is also possible that cognitive changes in UCSF's predominantly healthy or mildly symptomatic cohort reflect contributions of inflammation and disrupted synaptic signaling, which may be captured by rising plasma GFAP and precede neuronal loss.^49^, ^50^, ^51^, ^52^

Astrocyte reactivity may catalyze Aβ effects on early tau pathology, which in turn is associated with neurodegeneration.^11^ In line with this hypothesis, plasma GFAP also moderated the association of plasma p‐tau181 (considered a measure of Aβ‐induced tau phosphorylation^3^) and neurodegeneration, but only in the 1FLADRC cohort. This may relate to frank neurodegeneration, representing a later stage of disease severity, and the 1FLADRC having a higher frequency of cognitively impaired participants. Post hoc analyses suggested this effect in 1FLADRC was driven by Hispanic White participants, who were not as well represented in UCSF. There may be unique social or health exposures disproportionately contributing to inflammation in Hispanic White participants.^53^, ^54^, ^55^

Astrocyte reactivity estimated by plasma GFAP represents a non‐invasive, accessible modality with significant clinical implications for prognosis and as a therapeutic target for AD. Since higher levels of plasma GFAP tended to be associated with worse AD pathology, interventions and lifestyle activities modulating astrocyte reactivity may be protective against AD progression.^56^, ^57^ Plasma GFAP may also be useful for enriching AD clinical trials. Beyond Aβ alone, plasma GFAP may provide additional prognostic information for identifying individuals at greatest risk for imminent disease progression and cognitive decline. This is especially relevant for trials targeting early or preclinical disease populations^58^, ^59^, ^60^ where there is significant variability in the likelihood or timing of cognitive decline, especially when based on biomarkers like Aβ‐PET.^61^, ^62^, ^63^ Our preliminary post hoc findings suggest that plasma GFAP moderation of some components of AD pathobiology differs by sex and ethnoracial group. Plasma GFAP may increase more rapidly in females compared to males at early disease stages, such as those with preclinical AD.^64^ Given the clinical and treatment implications of identifying factors that might accelerate AD onset and progression, there remains a critical need to replicate and further investigate reasons for sex‐ or other demographic‐specific variability in the influence of astrocyte reactivity on AD.

### 4.1 Strengths and limitations

A strength of our study is the multicohort design spanning demographically and clinically diverse groups across the AD and cognitive continua. Our harmonized cognitive composites enabled robust cross‐cohort comparisons and may better capture early disease cognitive effects than CDR. Findings in 1FLADRC's majority Hispanic/Latino, cognitively impaired cohort complemented those in UCSF's majority non‐Hispanic/Latino White, cognitively unimpaired cohort. While consistent cross‐cohort results support the generalizability of findings, inconsistent findings could reflect cohort differences. Furthermore, findings in 1FLADRC's predominantly Cuban and South American participants may not generalize to other Hispanic/Latino groups (e.g., Mexican American). Our cross‐sectional design limits temporal inferences between AD biomarkers and cognitive decline, which would be optimally addressed with longitudinal data. Our models combined imaging and fluid AD biomarkers – future studies using consistent modalities (e.g., all imaging‐based or fluid‐based) as well as more direct tau measures (e.g., tau‐PET, biofluid MTBR‐243) could refine and contextualize our findings.^3^, ^65^, ^66^

## 5 CONCLUSIONS

Plasma GFAP provides a minimally invasive, accessible means for assessing astrocyte reactivity and appears to significantly moderate amyloid–tau associations across the AD spectrum and within demographically distinct cohorts. Astrocyte reactivity may more prominently and consistently influence earlier AD pathology than downstream effects on neurodegeneration and cognitive decline. There is growing evidence that plasma GFAP complements core AD biomarkers to inform the risk of disease progression.

## CONFLICT OF INTEREST STATEMENT

Authors report no disclosures relevant to the content of this study. Dr. Armstrong reported grants from the National Institutes of Health (NIH), Florida Department of Health, and Lewy Body Dementia Association Research Center of Excellence; speaker honoraria from the Taiwan International Congress of Parkinson's Disease and Movement Disorders, American Academy of Neurology Annual Meetings, World Congress on Parkinson's Disease and Related Disorders, PRIME CME Program, Dr. Daniel I. Kaufer Lecture Series at the University of Wisconsin Alzheimer's Disease Research Center, Dementia with Lewy Bodies: Filling the Gaps in Translational and Clinical Research NIA‐NINDS Conference, and Michael J. Fox Foundation Neuro‐Impact Workshop; personal compensation for serving as a Data Safety Monitoring Board member with the Alzheimer's Therapeutic Research Institute/ Alzheimer's Clinical Trials Consortium, Alzheimer's Disease Cooperative Study, and an NIH study (R01AG083828); and a non‐compensated relationship as a member of the Scientific Advisory Board Executive Committee for the Lewy Body Dementia Association. Dr. Casaletto reported grants from the National Institute on Aging (NIA), Hillblom Foundation, Alzheimer's Association, and Wellcome Trust, Leap; and non‐compensated relationships as Chair of the International Neuropsychological Society Conflict of Interest Committee and Executive Board member of the Alzheimer's Association ISTAART Cognition Professional Interest Area. Dr. DeKosky reported royalties from UpToDate as the section editor for dementia; consulting fees from Brainstorm Cell Therapeutics, Eisai, and Sanofi; personal fees from Acumen Pharmaceuticals, Biogen, Prevail Pharmaceuticals, Cognition Therapeutics, Vaccinex, Lilly Pharmaceuticals, Nido Biosciences, Neuvivo Pharmaceuticals, Novo Nordisk, and Capricor for serving on the data safety monitoring or medical advisory boards; and honoraria from *Neurotherapeutics* for serving as an associate editor and UpToDate for serving as a section editor for dementia outside the submitted work. Dr. DeSimone reported consulting fees from Automated Imaging Diagnostics; and being a shareholder for Automated Imaging Diagnostics. Dr. La Joie reported grants from the National Institute on Aging, the Alzheimer's Association, the US Department of Defense; consulting fees from GE Healthcare outside the submitted work; and conference attendance support from the Alzheimer's Association Dr. Rabinovici reported grants from National Institutes of Health during the conduct of the study; consulting fees from C2N, Eli Lilly, Alector, Merck, Roche, and Novo Nordisk; data safety monitoring board fees from Johnson & Johnson; and grants from Avid Radiopharmaceuticals, GE Healthcare, Life Molecular Imaging, and Genentech outside the submitted work. Dr. Rojas reported serving as site principal investigator for clinical trials sponsored by Eli Lilly, Eisai, and Amylyx during the conduct of the study; consulting fees from Ferrer International, AdeptField Solutions, Reach Market Research, and Clarivate; honoraria for lectures through the American Academy of Neurology; conference attendance support from the Alzheimer's Association; and a pending patent. Dr. Paolillo reported a grant from the NIA. Dr. Saloner reported grants from the Alzheimer's Association, New Vision Research Charleston Conference on Alzheimer's Disease, American Academy of Neurology, American Brain Foundation, and Association for Frontotemporal Degeneration. Dr. Smith reported a grant from the National Institute on Aging (NIA). Dr. Staffaroni reported personal fees from ADDF, Alector, Aviado Bio, CervoMed, Passage Bio, Prevail Therapeutics/Eli Lilly, Takeda, and Vesper Bio. Dr. Vaillancourt reported grants from the NIH; Neuropacs Corp shareholder; and licensed patents (11439341; 10758170). Dr. VandeVrede reported grants from the NIH, Alzheimer's Association, and Shenandoah Foundation; consulting fees from Roche, Siemens, and Biogen; personal fees from Peerview CME and Haymarket; payment for expert testimony; meeting attendance/travel support from Biogen, Siems, and Tau Consortium; serving on a monitoring or advisory board for NIO‐SILK; and receipt of materials or services from LabCorp and Quanterix outside the submitted work. Dr. Wiens reported a grant from the NIH. Dr. Wang reported a grant from the NIA. All other authors report no disclosures. The authors have no conflicts of interest. Author disclosures are available in the supporting information.

## CONSENT STATEMENT

All study participants provided informed consent prior to undergoing study procedures.

## Supporting information



Supporting Information

Supporting Information
