# Supplementary material for: Moderating effects of plasma glial fibrillary acidic protein along the Alzheimer's disease continuum
Source: Alzheimers Dement. 2025 Sep 5;21(9):e70626. doi: 10.1002/alz.70626 (PMC12412752; doi:10.1002/alz.70626)
Supplement: Supplementary file 1 — Supporting Information [file ALZ-21-e70626-s001.pdf]

Supplementary Methods

Plasma Collection and Processing

UCSF

Venous blood was collected in lavender-top EDTA-containing tubes and centrifuged at 1500g at 4°C for 15 minutes within 1 hour of collection. Plasma samples were stored at −80°C until analysis. Protein concentrations were quantified via single-molecule array (Simoa) for p-tau181 (pTau-181 Advantage V2, analyzed in 2021) and for GFAP and NfL (Simoa, Quanterix Neurology 4-Plex A). All analyses were performed in duplicate according to manufacturer protocols.

1FLADRC

Venous blood was collected in lavender-top EDTA tubes, gently mixed by inversion (10 times), and centrifuged at room temperature for 12 minutes at 1,200 rcf within one hour of collection. Plasma samples were stored at −80 °C until analysis. Protein concentrations were quantified via Simoa (Quanterix SR-X or HD-X) for p-tau181 (Advantage V2, analyzed in 2023) and for GFAP and NfL (Neurology 2-Plex B). Plasma p-tau217 (AlzPath) was quantified at the Quanterix Accelerator Laboratory (Quanterix, Billerica, MA) on the HD-X platform. All analyses were performed in duplicate according to manufacturer protocols.

MRI Acquisition and Processing

UCSF

Participants underwent structural magnetic resonance imaging (MRI) at the UCSF Neuroscience Imaging Center using either a Siemens Trio Tim or Prisma Fit 3T scanner. Magnetization prepared rapid gradient-echo (MPRAGE) sequences were used to obtain whole brain T1-weighted images sagittally using the following parameters: repetition time (TR) = 2300 ms, inversion time (TI) = 900 ms, echo time (TE) = 2.98 ms, flip angle = 9°, field-of-view (FOV) = 240×256 mm with 1×1 mm in-plane resolution and 1 mm slice thickness. Parameters for both Trio and Prisma scanners had nearly identical parameters but slightly different echo times (Trio: 2.98 ms; Prisma: 2.9 ms).

Before processing, all T1-weighted images were visually inspected for quality control and those with excessive motion or image artifact were excluded. Magnetic field bias was corrected using the N3 algorithm.[1] Tissue segmentation was performed using unified segmentation in SPM12,[2] with subsequent inspection to ensure robustness of the process. Each participant's native space gray matter segmentation was normalized and modulated, via nonlinear and rigid-body transformations, to study-specific template space using DARTEL-based VBM.[3] A Gaussian kernel of 4-mm full width half maximum was applied for smoothing of images. Transformations (linear and nonlinear) between DARTEL's space and ICBM space were conducted to enable statistical comparisons.[4] Brain volumes of interest were quantified by translating a standard parcellation atlas [5] into ICBM space and summing the gray matter within each region of interest (ROI).

1FLADRC

MRI scans were obtained on 3T Siemens scanners (Magnetom Skyra, Vida, Prisma) using a 20- or 64- channel head/neck coil. T1-weighted imaging was acquired using a magnetization-prepared rapid gradient-echo (MPRAGE) sequence with the following parameters: repetition time (TR) = 2300 ms, echo time (TE) = 2.95-2.98 ms, slices thickness =1.2 mm, gap = 0 mm, resolution = 1.1×1.1×1.2 mm³. For quality control, all raw T1 images were visually inspected for acquisition artifacts, including incomplete whole-brain coverage, magnetic field inhomogeneities and signal distortions, and overt pathological lesions.

T1 image processing was performed using an automated reconstruction pipeline in FreeSurfer (version 7.3.2: https://surfer.nmr.mgh.harvard.edu) which included skull stripping, intensity normalization, subcortical segmentation, cortical parcellation, and region-of-interest (ROI) labeling. FreeSurfer-based SBM was used in calculating cortical volume measures in 68 total ROIs (34 bihemispheric) using the Desikan–Killiany atlas.[5] Prior to analysis, reconstructed output files were visually inspected for cortical parcellation and subcortical segmentation errors.

Amyloid PET Acquisition and Processing

UCSF

A subset of participants (n=129) underwent Aβ-PET imaging with [^{18}F] florbetapir acquired on a GE Discovery STE/VCT PET-CT scanner at UCSF. As previously described,[6] Florbetapir acquisition and processing was conducted in accordance with the Alzheimer’s Disease Neuroimaging Initiative (ADNI) protocol. Participants were scanned from 50 to 70 min post-injection of 10 mCi of florbetapir. PET frames were smoothed and averaged to achieve an effective 8 × 8 × 8 mm resolution. Standard uptake value ratios (SUVR) were calculated using the whole cerebellum as the reference region and converted to a Centiloids (CL) scale, where “0” represents mean uptake in young, healthy controls without amyloid pathology and “100” corresponds with the typical degree of amyloid deposition observed in patients diagnosed with mild-to-moderate dementia due to Alzheimer’s disease.[7] Global amyloid status (+/-) was determined based on visual read. Participants completed Aβ-PET imaging within 365 days of blood draw.

1FLADRC

A subset of participants (n=264) completed Aβ-PET scans with [^{18}F] florbetaben (90.2%) or [^{18}F] florbetapir (9.8%) tracers acquired on Siemens scanners (Philip Gemini or Siemens Biograph). Acquisition parameters were used according to Standardized Centralized Alzheimer’s & Related Dementias Neuroimaging (SCAN) PET Technical Procedures Manual. A 20-min PET scan starting at least 90-110 min (florbetaben) or 50-70 min (florebetapir) after intravenous injection of radiotracers was conducted. The number of voxels was in the range of 120-128 (x, y dimension, voxel size 1mm – 2.6 mm) and 82-90 voxel (z dimension, 2 mm slice thickness). Images were smoothed with a 5 mm Gaussian filter. The amyloid PET scans were co-registered linearly with 6

degrees of freedom, onto subject's T1 weighted MPRAGE scan using FSL toolbox (fsl.fmrib.ox.ac.uk). The segmented MRIs from the FreeSurfer pipeline and the co-registered PET images were used to extract regional SUV by averaging the intensity of individual ROIs. SUVRs were computed by normalizing the regional SUV with the SUV from the whole cerebellum and transformed to Centiloids (CL). Following reconstruction, image sets were visually inspected, and global amyloid status (+/-) was determined based on visual read. All Aβ-PET scans were obtained within 365 days of blood draw.

Cognitive Assessment

In both study cohorts, our memory composite included Montreal Cognitive Assessment (MoCA) registration, immediate recall, and recognition, 10-minute visual delayed recall from the Benson Figure task and verbal immediate recall, verbatim UDS Craft Story immediate and delayed recall, and list learning sum of learning trials, long delay free recall, and recognition scores from the California Verbal Learning Test, second edition (CVLT-II, UCSF only) or Hopkins Verbal Learning Test (HVLT, 1FLADRC only).[8] For composite of executive functioning, we utilized the item response theory-based Uniform Data Set v3.0 EF composite score,[9] which included NACC Digit Span Backwards (total correct), Trail Making Test (TMT) parts A and B (correct lines per minute), lexical fluency (F and L words—total correct), and semantic fluency (animal and vegetable fluency—total correct) measures.

Supplementary Methods References

1. Sled JG, Zijdenbos AP, Evans AC. *A Nonparametric Method for Automatic Correction of Intensity Nonuniformity in MRI Data*. Vol 17.; 1998.

2. Ashburner J, Friston KJ. Unified segmentation. *Neuroimage*. 2005;26(3):839-851. doi:10.1016/j.neuroimage.2005.02.018

3. Ashburner J. A fast diffeomorphic image registration algorithm. *Neuroimage*. 2007;38(1):95-113. doi:10.1016/j.neuroimage.2007.07.007

4. Mazziotta JC, Toga AW, Evans A, Fox P, Lancaster J. A Probabilistic Atlas of the Human Brain: Theory and Rationale for Its Development. *Neuroimage*. 1995;2(2):89-101. doi:10.1006/nimg.1995.1012

5. Desikan RS, Ségonne F, Fischl B, et al. An automated labeling system for subdividing the human cerebral cortex on MRI scans into gyral based regions of interest. *Neuroimage*. 2006;31(3):968-980. doi:10.1016/j.neuroimage.2006.01.021

6. Saloner R, VandeVrede L, Asken BM, et al. Plasma phosphorylated tau-217 exhibits sex-specific prognostication of cognitive decline and brain atrophy in cognitively unimpaired adults. *Alzheimer's and Dementia*. 2024;20(1):376-387. doi:10.1002/alz.13454

7. Klunk WE, Koeppe RA, Price JC, et al. The Centiloid project: Standardizing quantitative amyloid plaque estimation by PET. *Alzheimer's and Dementia*. 2015;11(1):1-15.e4. doi:10.1016/j.jalz.2014.07.003

8. Sanderson-Cimino M, Gross AL, Gaynor LS, et al. Development and validation of a harmonized memory score for multicenter Alzheimer's disease and related dementia research. *medRxiv*. Published online January 1, 2025:2025.03.31.25324964. doi:10.1101/2025.03.31.25324964

9. Staffaroni AM, Asken BM, Casaletto KB, et al. Development and validation of the Uniform Data Set (v3.0) executive function composite score (UDS3-EF). *Alzheimer's and Dementia*. 2021;17(4):574-583. doi:10.1002/alz.12214

Supplementary Results

eTable 1. Aβ-PET sample participant characteristics by cohort

	UCSF	1FLADRC
**n**	129	264
**Age, years**	73.27 (7.4)	70.75 (7.9)
**Education, years**	17.23 (2.2)	15.8 (6.1)
**Sex, N (% Female) = 2 (%)**	64 (49.6)	149 (56.4)
**Ethno-Racial Group, N (%)**		
**Non-Hispanic/Latino White**	119 (92.2)	115 (43.6)
**Hispanic/Latino White***	N/A	128 (48.5)
**Black/African American**	2 (1.6)	15 (5.7)
**Other/Not Reported**	8 (6.2)	6 (2.2)
**APOE ε4, N (% Carrier)**	44 (34.1)	101 (38.3)
**Global CDR, N (%)**		
**0**	96 (74.4)	66 (25.0)
**0.5**	33 (25.6)	156 (59.0)
**1+**	0 (0.0)	42 (16.0)
**Aβ-PET (+), N (%)**	36 (27.9)	107 (40.5)
**Cognitively Unimpaired**	15 (41.7)	13 (12.1)
**Mild Cognitive Impairment**	21 (58.3)	62 (58.0)
**Dementia**	0 (0.0)	32 (29.9)
**Aβ-PET Centiloids (CL)**	21.3 (40.8)	29.5 (38.6)

Note: all values are reported as mean (SD), unless otherwise noted; SD = standard deviation

Abbreviations: APOE, apolipoprotein E; CDR, Clinical Dementia Rating scale

*Hispanic/Latino White participants in the 1FLADRC Aβ-PET sample (n= 128) reported the following ethnic origins: 68(53.1%) Cuban, 42(32.8%) South American, 10(7.8%) Puerto Rican, 8(6.3%) Other. Hispanic/Latino ethnic origin information was not available for the UCSF cohort.

eTable 2. Correlations between plasma GFAP and AT_1(N) biomarkers and cognition by study cohort

Correlation	UCSF	1FLADRC
**A_{CL} –GFAP**	0.38 [0.22, 0.52]***	0.34 [0.23, 0.44]***
**T_1 –GFAP**	0.59 [0.43, 0.74]***	0.48 [0.40, 0.56]***
**N_{MRI} –GFAP**	-0.38 [-0.50, -0.25]***	-0.32 [-0.41, -0.23]***
**N_{Plasma} –GFAP**	0.58 [0.47, 0.68]***	0.59 [0.51, 0.65]***
**Cog_{memory} –GFAP**	-0.21 [-0.35, -0.067]**	-0.21 [-0.31, -0.11]***
**$Cog_{executive}$–GFAP**	-0.16 [-0.35, 0.041]	-0.18 [-0.28, -0.80]***

Note: data shown as Pearson correlation coefficients (r) with 95% confidence intervals.
* p < .05, ** p < .01, *** p < .001

eTable 3. Main effects of A/T_1/N/Cog relationships by study cohort

Unconditional Model	UCSF	1FLADRC
**A_{VR} –T_1**	0.55 [0.40, 0.71]***	0.54 [0.44, 0.65]***
**A_{CL} –T_1**	0.59 [0.43, 0.74]***	0.60 [0.50, 0.71]***
**T_1 –N_{MRI}**	-0.13 [-0.24, -0.02]*	-0.26 [-0.34, -0.18]***
**T_1 –N_{plasma}**	0.23 [0.11, 0.36]***	0.41 [0.34, 0.49]***
**T_1 –Cog_{memory}**	-0.22 [-0.37, -0.07]**	-0.27 [-0.35, -0.18]***
**T_1 –$Cog_{executive}$**	-0.15 [-0.35, 0.05]	-0.20 [-0.28, -0.11]***
**N_{MRI} –Cog_{memory}**	0.45 [0.23, 0.66]***	0.52 [0.41, 0.63]***
**N_{MRI} –$Cog_{executive}$**	0.61 [0.33, 0.89]***	0.38 [0.27, 0.49]***
**N_{plasma} –Cog_{memory}**	-0.11 [-0.28, 0.05]	-0.19 [-0.28, -0.10]***
**N_{plasma} –$Cog_{executive}$**	0.03 [-0.18, 0.25]	-0.18 [-0.27, -0.09]***

Note: data shown as standardized betas with 95% confidence intervals.

* p < .05, ** p < .01, *** p < .001

eTable 4. Plasma GFAP moderation of A/T_1/N/Cog relationships by study cohort

GFAP<br>Interaction Model	UCSF	J-N GFAP Thresholds /<br>GFAP Main Effects	1FLADRC
**A_{VR} –T_1 x GFAP**	0.46 [0.10, 0.81]*	GFAP > 97.72	0.12 [0.01, 0.22]*
**A_{CL}–T_1 x GFAP**	0.16 [-0.01, 0.32]	GFAP > 93.32	0.13 [0.03, 0.23]*
**T_1 –N_{MRI} x GFAP**	-0.01 [-0.11, 0.10]	-0.14 [-0.26, -0.02]*	-0.09 [-0.18, 0.00]*
**T_1 –N_{plasma} x GFAP**	-0.03 [-0.13, 0.07]	0.43 [0.31, 0.56] ***	0.17 [0.11, 0.23]***
**T_1 –Cog_{memory} x GFAP**	-0.14 [-0.28, 0.00]*	GFAP > 173.78	-0.04 [-0.13, 0.04]
**T_1–$Cog_{executive}$ x GFAP**	-0.25 [-0.43, -0.07]**	GFAP > 245.47	0.0036 [-0.08, 0.08]
**N_{MRI} –Cog_{memory} x GFAP**	0.20 [0.06, 0.35]**	GFAP > 112.20	0.04 [-0.05, 0.13]
**N_{MRI} –$Cog_{executive}$ x GFAP**	0.19 [0.00, 0.39]*	GFAP > 100	-0.03 [-0.12, 0.07]
**N_{plasma} –Cog_{memory} x GFAP**	-0.07 [-0.19, 0.05]	-0.09 [-0.27, 0.09]	-0.01 [-0.08, 0.06]
**N_{plasma} –$Cog_{executive}$ x GFAP**	-0.10 [-0.23, 0.04]	-0.04 [-0.26, 0.19]	0.0037 [-0.06, 0.06]

Note: data shown as standardized betas with 95% confidence intervals. Plasma GFAP threshold values are measured in pg/mL.
* p < .05, ** p < .01, *** p < .001

eTable 5. Plasma GFAP moderation of A/T_1/N/Cog relationships by sex, by study cohort

GFAP x Sex Interaction Model	UCSF	1FLADRC
**A_{VR} –T_1 x GFAP x Sex**	0.13 [-0.06, 0.32]	0.02 [-0.09, 0.14]
**A_{CL} –T_1 x GFAP x Sex**	0.09 [-0.09, 0.26]	-0.01 [-0.13, 0.10]
**T_1 –N_{MRI} x GFAP x Sex**	0.03 [-0.08, 0.14]	0.01 [-0.07, 0.10]
**T_1 –N_{plasma} x GFAP x Sex**	-0.03 [-0.08, 0.14]	0.09 [0.03, 0.16]**<br>Female: 0.26 [0.18, 0.34]***<br>Male: 0.07 [-0.03, 0.16]

**T_1 –Cog_{memory} x GFAP x Sex**	-0.02 [-0.17, 0.13]	-0.13 [-0.25, -0.01]*<br>Female: -0.12 [-0.24, 0.00]*<br>Male: 0.04 [-0.07, 0.16]
**T_1 –$Cog_{executive}$ x GFAP x Sex**	0.06 [-0.15, 0.26]	-0.01 [-0.09, 0.07]
**N_{MRI} –Cog_{memory} x GFAP x Sex**	0.01 [-0.18, 0.20]	0.04 [-0.06, 0.14]
**N_{MRI} –$Cog_{executive}$ x GFAP x Sex**	-0.10 [-0.37, 0.17]	0.04 [-0.06, 0.14]
**N_{plasma} –Cog_{memory} x GFAP x Sex**	0.02 [-0.11, 0.16]	-0.0040 [-0.08, 0.07]
**N_{plasma} –$Cog_{executive}$ x GFAP x Sex**	0.08 [-0.08, 0.24]	0.02 [-0.05, 0.08]

Note: data shown as standardized betas with 95% confidence intervals.
* p < .05, ** p < .01, *** p < .001

eTable 6. Plasma GFAP moderation of A/T_1/N/Cog relationships by ethno-racial group in 1FLADRC

GFAP x Ethno-Racial Group Interaction Model	1FLADRC
**A_{VR} –T_1 x GFAP x Ethno-Racial Group**	HW: 0.06 [-0.42, 0.55]
**A_{CL} –T_1 x GFAP x Ethno-Racial Group**	HW: -0.01 [-0.23, 0.21]
**T_1 –N_{MRI} x GFAP x Ethno-Racial Group**	HW: -0.04 [-0.26, 0.17]<br>AA: -0.24 [-0.49, 0.01]
**T_1 –N_{plasma} x GFAP x Ethno-Racial Group**	HW: 0.20 [0.05, 0.36]**<br>AA: 0.18[-0.02, 0.37]
**T_1 –Cog_{memory} x GFAP x Ethno-Racial Group**	HW: 0.11 [-0.10, 0.33]<br>AA: 0.04 [-0.20, 0.28]
**T_1 –$Cog_{executive}$ x GFAP x Ethno-Racial Group**	HW: -0.02[-0.21, 0.17]<br>AA: -0.11 [-0.36, 0.13]
**N_{MRI}–Cog_{memory} x GFAP x Ethno-Racial Group**	HW: -0.17 [-0.37, 0.03]<br>AA: 0.08 [-0.20, 0.36]
**N_{MRI} –$Cog_{executive}$ x GFAP x Ethno-Racial Group**	HW: -0.05 [-0.25, 0.15]<br>AA: 0.09 [-0.19, 0.36]
**N_{plasma} –Cog_{mem} x GFAP x Ethno-Racial Group**	HW: -0.09 [-0.27, 0.09]<br>AA: 0.03 [-0.15, 0.21]
**N_{plasma} –$Cog_{executive}$ x GFAP x Ethno-Racial Group**	HW: -0.10 [-0.25, 0.06]<br>AA: -0.08 [-0.26, 0.11]

Note: data shown as standardized betas with 95% confidence intervals.
* $p < .05$, ** $p < .01$, *** $p < .001$

eTable 7. Plasma p-tau217 replication of main effect and plasma GFAP moderation relationships in 1FLADRC

Model	1FLADRC p-tau217	J-N GFAP Thresholds
**A_{VR} –T_1**	0.61 [0.50, 0.71]***	N/A
**A_{VR}–T_1 x GFAP**	0.25 [0.06, 0.44]**	GFAP > 54.95
**A_{CL} –T_1**	0.66 [0.56, 0.76]***	N/A
**A_{CL} –T_1 x GFAP**	0.11 [0.01, 0.20]*	GFAP > 39.81
**T_1 –N_{MRI}**	-0.37 [-0.46, -0.28]***	N/A
**T_1 –N_{MRI} x GFAP**	-0.12 [-0.21, -0.03]*	GFAP > 89.13
**T_1 –N_{plasma}**	0.47 [0.39, 0.55]***	N/A
**T_1 –N_{plasma} x GFAP**	0.14 [0.07, 0.20]***	GFAP > 109.64
**T_1 –Cog_{memory}**	-0.40 [-0.49, -0.30]***	N/A
**T_1 –Cog_{memory} x GFAP**	-0.06 [-0.15, 0.03]	N/A
**T_1 –$Cog_{executive}$**	-0.25 [-0.35, -0.16]***	N/A
**T_1 –$Cog_{executive}$ x GFAP**	0.01 [-0.08, 0.09]	N/A

Note: data shown as standardized betas with 95% confidence intervals. Plasma GFAP threshold values are measured in pg/mL.
* p < .05, ** p < .01, *** p < .001
